# Thermal environment simulation and analysis of renovated solar greenhouses with enhanced cultivation area and space

**DOI:** 10.1371/journal.pone.0335954

**Published:** 2025-11-06

**Authors:** Jieran Liu, Dongshuai Liang, Yunfei Ma

**Affiliations:** Institute of Yantai, China Agricultural University, Yantai, China; Politecnico di Milano, ITALY

## Abstract

In response to the issues of low land utilization efficiency and poor nighttime thermal performance in old single-slope solar greenhouses (SSG) commonly found in northern China, this study proposes renovation measures that expand the cultivation area and interior space by adding a shaded room and lowering its ground level. These modifications transform the original SSG into a double-slope solar greenhouse (DSG) and a sunken double-slope solar greenhouse (SDSG). Computational Fluid Dynamics (CFD) was employed to simulate and analyze the thermal environments of the three greenhouse types. The results indicate that, in winter, the peak temperature of the sunlit side in the SDSG is 1.9°C higher than that in the SSG; the temperature of the shaded side in the SDSG is 0.88–1.81°C higher than that in the DSG; compared with the rear wall of the SSG, the heat flux density of the middle wall in the SDSG is 10.19 W/m² lower, and is similar to that of the DSG middle wall, but the duration of heat release is longer in the SDSG; in comparison to the SSG and DSG, the annual thermal stability index of the SDSG is improved by 70% and 8.5%, respectively.

## Introduction

Solar greenhouses are a common type of single-slope greenhouse widely used in northern China [1]. As of 2024, the total area of solar greenhouses exceeds 195,000 mu, among which over 118,100 mu are old solar greenhouses that have been in use for more than ten years and face issues such as low land utilization efficiency and poor nighttime thermal performance [2–4]. In 2023, the Chinese government explicitly proposed a development policy for protected agriculture that focuses on upgrading and renovating outdated facilities, with the primary goal of improving the utilization efficiency of agricultural resources such as sunlight, heat, water, and soil, as well as the input-output efficiency of various production factors [5]. As a result, research on renovation technologies for old solar greenhouses has become a focal point of attention.

Wu et al. [6] improved the heat absorption capacity of single-slope solar greenhouses by adding an additional transparent roofing layer to the exterior of the structure. Wang et al. [7] upgraded solar greenhouses by reducing wall thickness and adjusting frame angles, thereby enhancing structural stability. Zhou Ying [8] conducted comparative experiments on solar greenhouses with different insulation walls and found that walls composed of a mixture of soil and polystyrene panels exhibited superior insulation and heat storage performance. Qu et al. [9] proposed an energy storage device installed on the north wall of the greenhouse to regulate light and thermal conditions, which effectively alleviated the problem of low nighttime indoor temperatures. Xia et al. [10] evaluated the thermal performance of traditional brick walls and new composite walls, clarifying the advantages of the latter in heating and insulation, thus improving the overall insulation of solar greenhouses. Liu et al. [11] compared the heat storage and release capacities of different types of north walls, designing a more reasonable inner surface structure for the north wall to further enhance the thermal performance of solar greenhouses. Ma et al. [12] designed a novel roofing material and structure for the shaded side of double-slope solar greenhouses, thereby enhancing the thermal environment of the shaded area. Liu et al. [13] upgraded the thermal environment of old single-slope solar greenhouses by replacing the plastic film lighting surface with glass and improving the performance of insulation materials. Although these studies have enhanced the insulation and thermal performance of solar greenhouses, the fundamental problem of low land utilization efficiency remains unresolved. Zhou et al. [14] proposed a method in which the rear wall of a traditional single-slope solar greenhouse is used as a shared wall, and an additional single-slope greenhouse facing north is constructed on the north side of this wall. The southern and northern single-slope greenhouses thus form a double-slope solar greenhouse with a central shared wall; the southern greenhouse is referred to as the sunlit side, and the northern as the shaded side. While this renovation method effectively increases land utilization efficiency, the working height of the shaded side is limited by the original height of the rear wall, making it unsuitable for practical agricultural production. Yu et al. [15] also investigated the thermal environment performance of a new type of double-slope solar greenhouse, concluding that it offers reduced air temperature fluctuations and significantly improved thermal regulation. However, the recessed roof design of this new double-slope solar greenhouse can lead to rain and snow accumulation, posing risks of leaks and excessive roof loads [16].

CFD technology is an effective tool for simulating and evaluating the thermal environment of solar greenhouses [17,18]. Tong et al. [19] used CFD to analyze the influence of different span widths on the temperature environment within solar greenhouses, and established proportional temperature variation patterns. Zhang et al. [20] employed CFD and weighted entropy to simulate and optimize the temperature distribution in solar greenhouses in China, identifying optimal energy-saving boundary conditions for practical application. Ma et al. [21] used CFD to simulate the insulation effects of composite walls and brick walls in solar greenhouses, concluding that block composite walls exhibit significantly superior thermal performance compared to brick walls. Liu et al. [22] applied CFD to evaluate the performance of three widely used radiation models in solar greenhouses, identifying the respective advantages of each model. Zhang et al. [23] constructed a large-span greenhouse model based on CFD and, by simulating the temperature and airflow field under natural ventilation, verified that the numerical simulation results agreed well with measured data. Wang et al. [24] studied the microclimate of a typical plastic greenhouse widely used in central China using a CFD model and demonstrated the model’s feasibility. Saberian et al. [18] used CFD to predict and analyze long-term dynamic microclimates, verifying the accuracy and reliability of the predictions. He et al. [25] investigated the effect of rear wall ventilation opening size on greenhouse cooling through CFD, concluding that a 1.4 m rear wall vent can improve ventilation efficiency. By utilizing CFD simulation, it is possible to predict and analyze changes in thermal environment parameters such as temperature and humidity under different renovation schemes before implementation, and to verify the feasibility of the results. This approach provides guidance for optimizing renovation plans, thereby reducing resource waste and minimizing the losses associated with unnecessary or misguided modifications [26–28].

Expanding the cultivation area and interior space to create a thermal environment suitable for crop growth is the primary objective of solar greenhouse renovation. Based on a typical old single-slope solar greenhouse (SSG) commonly found in northern China, this study introduces renovation measures such as adding a shaded room and lowering its ground level to form a double-slope solar greenhouse (DSG) and a sunken double-slope solar greenhouse (SDSG). Using CFD technology, the thermal environments of the three types of solar greenhouses were simulated and analyzed, and the results provide theoretical support and a reference basis for the renovation of old SSGs.

## Materials and methods

### Construction of renovation models

**Fig 1:**
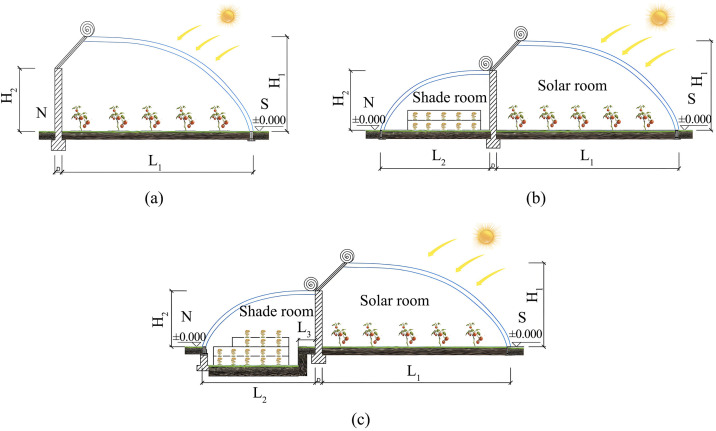
Structural configurations of the three types of solar greenhouses. (a) a. Cross-sectional view of a single-slope solar greenhouse (b) Cross-sectional view of a double-slope solar greenhouse (c) Cross-sectional view of a sunken double-slope solar greenhouse. Note: S denotes the south side, and N denotes the north side.

A typical old single-slope solar greenhouse (SSG) commonly found in northern China was selected as the subject for renovation, with its cross-sectional form shown in Fig 1a. Based on the existing SSG, a shaded section was added utilizing the original rear wall. Calculations indicate that the land utilization rate of the old SSG is only 41.5%. After the addition of the shaded section, the land utilization rate increases to 76.9%, representing an improvement of 35.4% over the original configuration [29].

For existing groups of SSGs, if the shaded room is built to the same height as the sunlit room, it will cast shadows on the rear row of greenhouses and cause rain and snow accumulation on the roof. Therefore, the configuration shown in Fig 1b is adopted to form a DSG, thereby increasing the cultivation area of the greenhouse. However, due to the height limitation of the original rear wall, the working height of the shaded room in the DSG is insufficient for practical agricultural operations. According to the traditional practice in northern China of excavating 0.8–1.2 meters below ground for sunken single-slope solar greenhouses [30], and taking into account the stability of the SSG rear wall foundation, the ground level of the shaded room in the DSG was excavated by 1 meter to form an SDSG, as shown in Fig 1c. By increasing the soil contact area and volume, this renovation further expands the cultivation space of the shaded room in the double-slope solar greenhouse, while simultaneously ensuring proper greenhouse spacing, natural lighting, and adequate working space in the shaded room. The dimensions of the greenhouses before and after renovation are listed in Table 1.

**Table 1:** Materials and Dimensions of the Solar Greenhouses.

Solar Greenhouse	Length	Span of Sunlit Section (L1)	Span of Shaded Section (L2)	Ridge Height of Sunlit Section (H1)	Ridge Height of Shaded Section (H2)	Wall Thickness (D)	Shaded Section Walkway (L3)
SSG	60m	10m	0	4.5m	0	370mm	0
DSG	60m	10m	6m	4.5m	3m	370mm	0
SDSG	60m	10m	6m	4.5m	3m	370mm	0.9m

### Research methods

Field tests were conducted on the DSG, and the differences between the measured data and simulation results were used to validate the accuracy of the CFD model. CFD simulations were then carried out to analyze the thermal environments of the SSG, DSG, and SDSG under identical boundary conditions, as well as the heat storage and release characteristics of the central wall. The results obtained were discussed and compared.

#### Experimental greenhouse.

**Fig 2:**
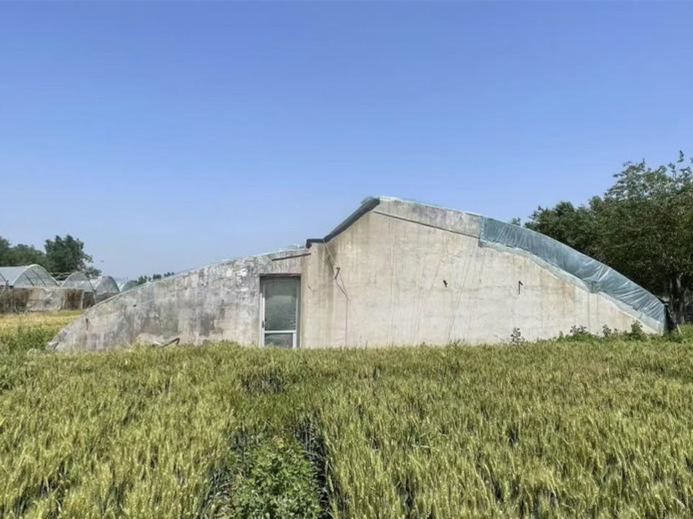
Overall exterior view of the experimental solar greenhouse.

The experiment was conducted in Jinan City, Shandong Province, China(36°40′N, 117°00′E), with the permission of Shandong Tingwang Agricultural Technology Co., Ltd. The experimental greenhouse(as shown in Fig 2) faces south with its back to the north, has a total length of 60 meters, a sunlit section span of 10 meters, and a shaded section span of 6 meters. The rear wall is 3 meters high, with the rear and gable walls being 0.37 meters thick, all constructed from red bricks. The greenhouse roof is covered with a 0.0001-meter-thick polyvinyl chloride (PVC) film, and no crops were planted inside during the experiments.

#### Experimental equipment and methods.

The experimental monitoring parameter was temperature. A total of 27 temperature measurement points were set up, each equipped with a temperature probe. Temperature monitoring was conducted using Apresys 179-THL temperature and humidity data loggers, with a measurement range of −30–70°C and an accuracy of ±0.2°C.

**Fig 3:**
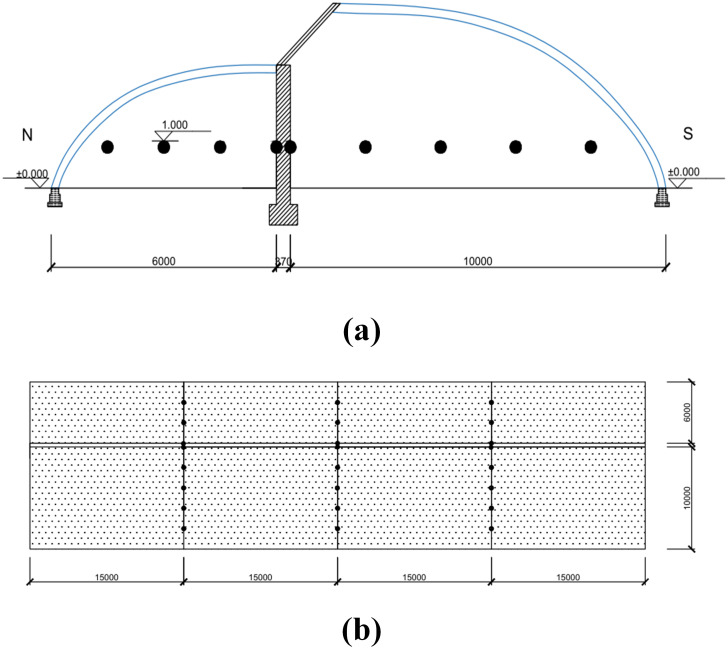
Layout of temperature monitoring points in the experimental double-slope solar greenhouse. (a) Cross-sectional layout of temperature monitoring points (b) Plan Layout of temperature monitoring points. Note: ● indicates the locations of temperature monitoring points. Dimensions are marked in millimeters (mm).

The arrangement of the temperature measurement points is shown in Fig 3:

1) Sunlit Section: The main temperature monitoring plane is the cross-section at the midpoint (1/2) of the greenhouse length. Starting from the rear wall and moving southward, a monitoring point is placed every 2 meters in the north-south direction, for a total of five measurement points. In addition, two other cross-sections located 15 meters east and west of the midpoint are selected, with measurement points arranged in the same manner.
2) Shaded Section: The main temperature monitoring plane is the cross-section at the midpoint (1/2) of the greenhouse length. Starting from the rear wall and moving northward, a monitoring point is placed every 1.5 meters in the north-south direction, for a total of four measurement points. Similarly, two other cross-sections located 15 meters east and west of the midpoint are selected, with measurement points arranged in the same manner.
3) All measurement points are positioned 1 meter above the ground.

**Fig 4:**
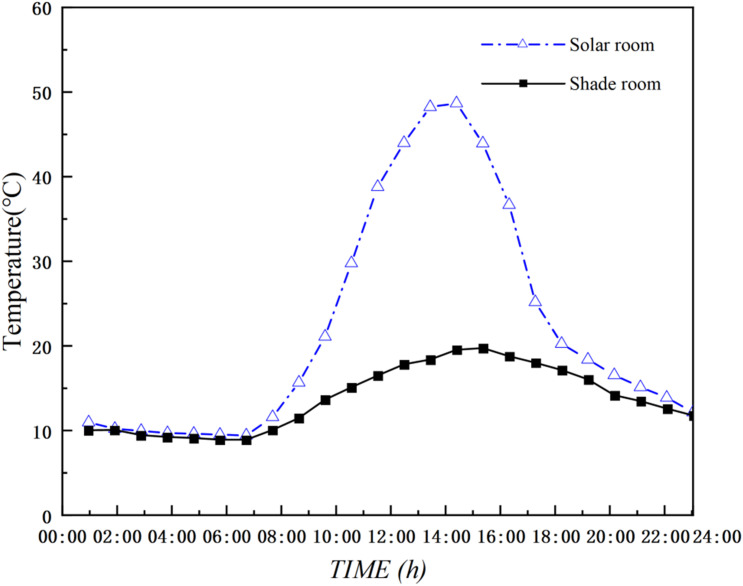
Full-day temperature measurements of the experimental dsg.

The experiment was conducted from March 20 to March 22, 2025. Temperature monitoring was set to record data every 5 minutes. The data in Fig 4 represent the arithmetic average of the 15 measurement points in the sunlit room and the 12 measurement points in the shaded room, reflecting the overall temperature variation trend of the DSG. Testing revealed that the arithmetic average of the measurement points at the central section of the greenhouse (at 30 m) was essentially consistent with the data presented in Fig 4 (S1 File).

## Model construction and boundary condition settings

### Solution method

The simulation was performed using Fluent software (ANSYS). The settings included a total of 2,880 time steps, each with a step size of 30 seconds, and 20 iterations per step. During the simulation process, a model was established in the CFD software based on the parameters of the experimental greenhouse, with mesh division, boundary condition settings, initial condition settings, simulation, and data processing carried out accordingly. The temperature data and distribution obtained from the experiment were compared with those generated by the CFD simulation to validate the results and analyze the thermal environment variations of different types of solar greenhouses.

### Model simplifications

1) The model does not consider the influence of crops inside the greenhouse on the thermal environment.
2) It is assumed that the simulation is conducted in a sealed environment, with no indoor ventilation or air exchange.
3) To further simplify the physical model, and according to the previous research by Yu et al. [31], the propagation range of soil temperature is significant within 15 cm and must be considered in the calculations; beyond 1 meter, the temperature gradient essentially disappears and can be neglected. Therefore, in the model, the soil boundary is set to 1 meter, and any heat transfer beyond this range is considered negligible.

### Physical model

**Fig 5:**
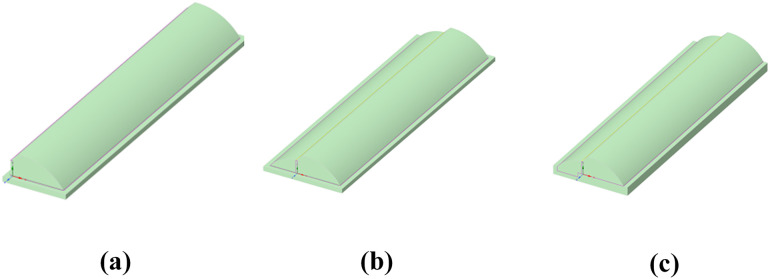
Three types of solar greenhouse models. (a) Single-Slope Solar Greenhouse (b) Double-Slope Solar Greenhouse (c) Sunken Double-Slope Solar Greenhouse.

To facilitate simulation analysis, the model was simplified as much as possible without compromising the authenticity or required accuracy of the simulation. Based on the Geometry module in Ansys Fluent, three-dimensional models of the three types of solar greenhouses were constructed, as shown in Fig 5. The positive direction of the Z-axis is west, and the positive direction of the X-axis is south. The three types of solar greenhouses use the same enclosure materials and parameters, as listed in Table 2. Structural dimensions are given in Table 1, and the soil boundary extends 1 meter outward in both the horizontal and vertical directions.

**Table 2:** Properties of construction materials [32].

Material	Density (kg·m^-3^)	Specific Heat Capacity (J·kg^-1^·°C^-1^)	Thermal Conductivity (W·m^-1^·°C^-1^)	Refractive Index
Air	1.225	1 006.43	0.0242	—
Red Brick	1700	840	0.42	—
Soil	1400	1100	0.94	—
Plastic Film	920	2100	0.30	1.48
Thermal Insulation Quilt	300	1275	0.11	—

Mesh generation was performed using Fluent Meshing to create hexahedral meshes, with a basic grid size of 0.15 meters for the entire computational domain. The total number of mesh cells generated was 1.56469 × 10^6^, 2.45760 × 10^6^, and 3.07869 × 10^6^ for the respective models. Mesh quality was assessed using skewness as the distortion criterion, with maximum values of 0.434, 0.497, and 0.537, and average values of 0.009, 0.009, and 0.008, respectively. All three greenhouse models met the mesh quality standards upon verification, indicating good overall mesh quality.

### Governing equations

#### Conservation equations for fluid flow.

When simulating air as an incompressible fluid and applying the fundamental physical conservation laws of mass, momentum, and energy for fluid motion, a set of coupled conservation equations can be used to obtain various thermal environment parameters within the computational domain. The continuity equation, momentum equation, and energy equation [33] are as follows:


$$\frac{\partial (\rho \varphi_{B})}{\partial t}+div(\rho \vec{\nu }\varphi_{B})=div(\Gamma grad\varphi_{B})+S$$ (1)

In these equations, *φ_B_* is a general variable that may represent any of the solved variables, such as *u*, *v*, or *t*. Where, *u*, *v*, and *t* represent the velocity components in three directions (m/s); *ρ* is the material density (kg/m^3^); *Γ* is the generalized diffusion coefficient; and *S* is the generalized source term. The terms in the equation correspond to, in order: the transient term, the convection term, the diffusion term, and the source term.

#### Turbulence model.

Due to the high calculation accuracy, broad applicability, and strong computational stability of the standard k-ε turbulence model—along with its moderate computational demand [34]—this model was adopted in this study. The standard k-ε model determines the turbulence length scale ε and the time scale by solving two separate transport equations, where k represents the turbulent kinetic energy and ε denotes the turbulent kinetic energy dissipation rate. The governing equations [35] are as follows:

k Equation:


$$\rho \frac{\partial k}{\partial t}+\rho u_{j}\frac{\partial k}{\partial x_{j}}=\frac{\partial }{\partial x_{j}}\left[\left(\eta +\frac{\eta_{t}}{\sigma_{k}}\right)\frac{\partial k}{\partial x_{j}}\right]+\eta_{t}\frac{\partial u_{i}}{\partial x_{j}}\left(\frac{\partial u_{i}}{\partial x_{j}}+\frac{\partial u_{j}}{\partial x_{i}}\right)-\rho \varepsilon$$ (2)

ε Equation:


$$\rho \frac{\partial \varepsilon }{\partial t}+\rho u_{k}\frac{\partial \varepsilon }{\partial x_{k}}=\frac{\partial }{\partial x_{k}}\left[\left(\eta +\frac{\eta_{t}}{\sigma_{\varepsilon }}\right)\frac{\partial \varepsilon }{\partial x_{k}}\right]+\frac{c_{\text{1}}\varepsilon }{k}\eta_{t}\frac{\partial u_{i}}{\partial x_{j}}\left(\frac{\partial u_{i}}{\partial x_{j}}+\frac{\partial u_{j}}{\partial x_{i}}\right)-c_{\text{2}}\rho \frac{\varepsilon^{\text{2}}}{k}$$ (3)

In these equations: *x*, *y*, and *z* represent the positive directions of the three axes in the spatial coordinate system; *μ* is the dynamic viscosity; *ρ* is the density; c_μ_ = 0.09, c_1_ = 1.44, c_2_ = 1.92, σ_k_ = 1.0, and σ_ε_ = 1.3.

#### Radiation model.

The solar radiation intensity was modeled using the Discrete Ordinates (DO) radiation model in Fluent. The radiation intensity was calculated based on the latitude and longitude of Jinan, China, as well as the local time. Solar position tracking was applied to enable transient simulation of solar radiation. The governing equation [36] is as follows:


$$\nabla \cdot [I(r,s)]+(\alpha +\sigma_{s})\cdot I(r,s)=\alpha \cdot n^{\text{2}}\cdot \frac{\sigma \cdot T^{\text{4}}}{4\pi }\int_{\text{0}}^{4\pi }\;\cdot I(s,s^{\prime })\cdot \varphi (r\cdot s)d\overset{´}{\Omega }$$ (4)

Where, r is the position vector; s denotes the direction vector; a is the absorption coefficient; σ_S_ is the scattering coefficient; I represents the radiation intensity (W/m^2^); n is the coefficient of refraction; T is the temperature (°C); φ is the phase function; and denotes the solid angle (180^2^/π^2^).

### Boundary and initial conditions

The boundary conditions primarily involve the thermal variables at the boundaries. According to the assumptions, there is no indoor ventilation during the simulation. The greenhouse envelope is set as a 370 mm thick brick wall. The convective heat transfer coefficient at the surface is calculated [37] as follows:


$$\alpha_{ci}=2.5+4.2V$$ (5)

$$\alpha_{co}=(2.5\sim 6.0)+4.2V$$ (6)

Where, α_ci_ and α_co_ represent the heat transfer coefficients at the inner and outer surfaces, respectively, in units of W/(m^2^ ·°C); V denotes wind speed in m/s; (2.5–6.0) is the range of constant values accounting for the effect of natural convection. Considering natural convection between the ground and the outside air, the heat transfer coefficient for the simulation in Jinan was set to 5 W/(m^2^ ·°C).

The actual temperatures measured at the experimental monitoring points were used as the initial temperatures for each greenhouse in the CFD simulations. The simulation period spanned from 00:00 on March 20, 2025, to 00:00 on March 22, 2025. It should be noted that, since no thermal insulation quilts were installed outside the test greenhouse, model validation was conducted under conditions without insulation, to match the actual situation. However, in subsequent simulation analyses reflecting real production scenarios, thermal insulation quilts were installed on the roofs of all three types of greenhouses. For both the sunlit sections of the DSG and SDSG, the insulation quilt was rolled up from 9:00–16:00 and deployed from 16:00–9:00 the next day. Since the shaded sections are typically used to grow edible fungi, which, except during certain developmental stages requiring diffuse light for morphological stimulation, do not need direct sunlight during most growth stages, the insulation quilt for the shaded section of the DSG was kept in place over two-thirds of the area, leaving only the bottom third open to allow the entry of diffuse light and facilitate the opening and closing of the quilt.

### Model validation

**Fig 6:**
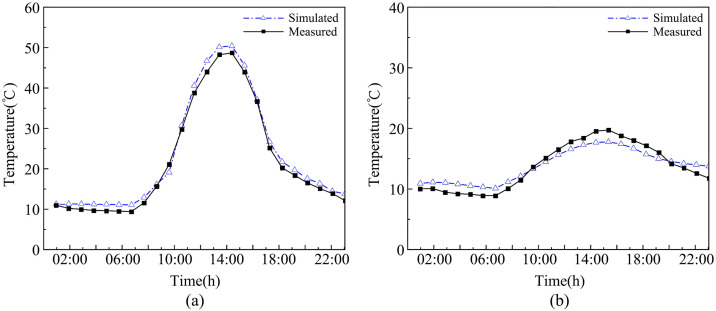
Comparison of simulated and measured indoor air temperatures in the double-slope solar greenhouse. (a) Comparison of Simulated and Measured Values for the Sunlit Room of the Double-Slope Solar Greenhouse (b) Comparison of Simulated and Measured Values for the Shaded Room of the Double-Slope Solar Greenhouse. Note: “Simulated” refers to the simulated values, “measured” refers to the experimental values.

To verify the accuracy of the CFD model, the simulated temperature results for both the sunlit and shaded sections of the DSG were compared with the measured experimental data, enabling an evaluation of the model’s precision. Both simulated and measured values were taken from the main monitoring cross-section at 30 m: for the sunlit room, the average of five measurement points on the section; and for the shaded room, the average of four measurement points on the section. As shown in Fig 6, the trends in the simulated and measured temperature curves for the sunlit and shaded sections of the DSG on March 21, 2025, were consistent. The average relative error was within ±1.32°C, with the maximum deviation reaching 1.98°C. The mean and maximum relative errors for the temperature field were 6.1% and 9.1%, respectively. For most fluid dynamics problems, an error within 10% is generally considered acceptable, indicating that the boundary condition settings under this meshing standard are valid and the results accurately reflect the environmental distribution within the greenhouse [38]. This demonstrates a good level of agreement for the model.

## Results validation and analysis

### Indoor temperature distribution in the greenhouse

**Fig 7:**
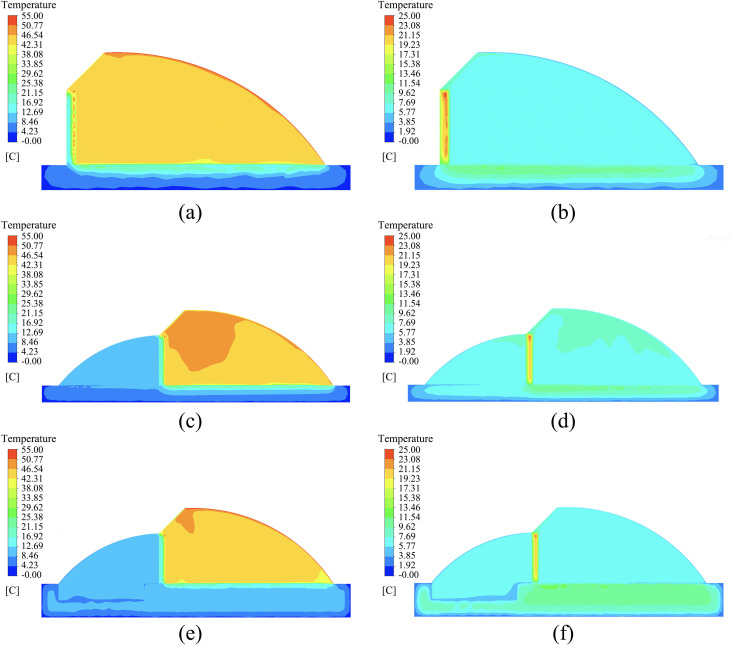
Simulated temperature fields of the three types of solar greenhouses at different times. (a) Temperature Field of the Single-Slope Solar Greenhouse at 14:00 (b) Temperature Field of the Single-Slope Solar Greenhouse at 24:00 (c) Temperature Field of the Double-Slope Solar Greenhouse at 14:00 (d) Temperature Field of the Double-Slope Solar Greenhouse at 24:00 (e) Temperature Field of the Sunken Double-Slope Solar Greenhouse at 14:00 (f) Temperature Field of the Sunken Double-Slope Solar Greenhouse at 24:00.

December 21 (the winter solstice) was selected as the date for analyzing indoor temperatures in the greenhouses, as outdoor climate conditions are typically more severe during this period, making it possible to effectively compare the temperature variation trends and differences among the three types of greenhouses. Since the length of the solar greenhouses is much greater than either the span or the height, the study focused on the temperature distribution field at the central cross-section, which is located 30 meters from each gable wall (east and west ends). The temperature fields at this central section for the three types of solar greenhouses at different time points are shown in Fig 7.

As shown in Fig 7, at 14:00, all three types of greenhouses exhibit a general downward temperature gradient with increasing height. The average sunlit room temperatures of the DSG and SDSG are 8.5% and 4.5% higher, respectively, than that of the SSG, while the differences in average temperatures within the shaded rooms are minimal. At 24:00, the walls and soil become the primary heat sources of the greenhouses, and the temperature gradient shifts to decrease from the rear wall and soil outward and into the interior. The average sunlit room temperatures of the DSG and SDSG are 11.5% and 1.3% higher, respectively, than that of the SSG, with little difference observed in the average shaded room temperatures.

#### Comparison of sunlit section temperature variations.

**Fig 8:**
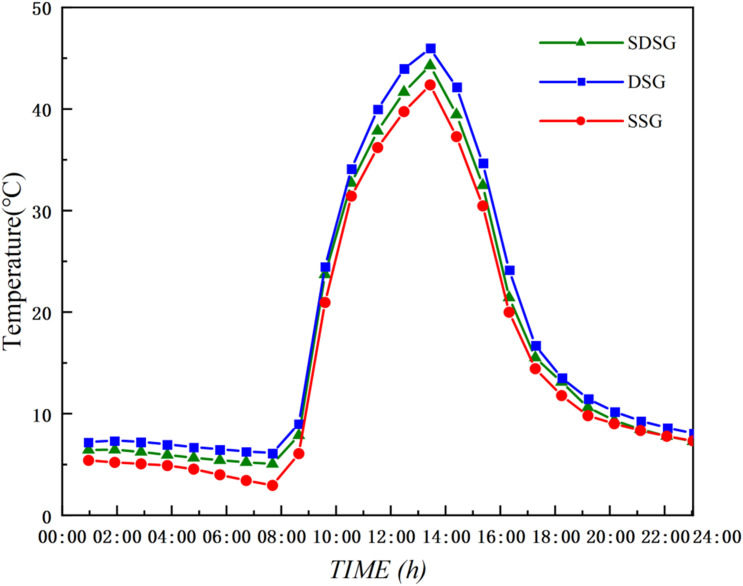
Comparison of simulated sunlit section temperatures throughout the day. Note: ssg stands for single-sided solar greenhouse, dsg stands for double-sided solar greenhouse, and SDSG stands for sunken double-sided solar greenhouse.

A comparative analysis was conducted on the temperature fields of the sunlit sections in the SSG, DSG, and SDSG, with the curves of average temperature values at different times throughout the day shown in Fig 8. The results indicate that the overall temperature curves of the sunlit sections in the SSG, DSG, and SDSG are generally consistent, displaying a parabolic trend. Notably, the diurnal temperature fluctuation of the indoor air in the sunken double-slope solar greenhouse is relatively smaller.

As shown in Fig 8 (S2 File), due to solar radiation intensity, the indoor temperatures of the three solar greenhouses reach their maximum values around 14:00. The maximum sunlit room temperature in the SDSG reaches 44.28°C, which is 1.9°C higher than the SSG maximum (42.38°C) and 0.61°C lower than the DSG maximum (39.84°C). Between 10:00 and 18:00, the indoor air temperature of the DSG sunlit room is higher than that of the SDSG. This is because the shaded room of the SDSG is larger than that of the DSG, and the sunken shaded room acts as a greater cold source during the day, requiring more heat transfer from the sunlit room to maintain the indoor environment. As a result, extreme high temperatures in the sunlit room are suppressed, and the internal heat distribution of the greenhouse is optimized. In contrast, due to the relatively poor insulation performance of its rear wall, the SSG maintains a slightly lower average indoor temperature compared to the sunlit rooms of both the DSG and SDSG.

From 18:00–9:00 the next day, the temperature curves of the three solar greenhouses display similar trends, with the temperatures in the sunlit rooms of the SSG, DSG, and SDSG all dropping significantly. Among them, the minimum nighttime temperature of the DSG sunlit room is 6.13°C, while that of the SDSG sunlit room is 5.02°C. Compared with the SSG minimum nighttime temperature of 2.94°C, these values are higher by 1.11°C and 3.19°C, respectively. This is because the shaded rooms of both the DSG and SDSG effectively form an insulation layer composed of the shaded roof and shaded space, which reduces heat loss from the rear wall of the sunlit room, thereby helping to maintain higher temperatures inside the sunlit room.

#### Comparison of shaded section temperature variations.

**Fig 9:**
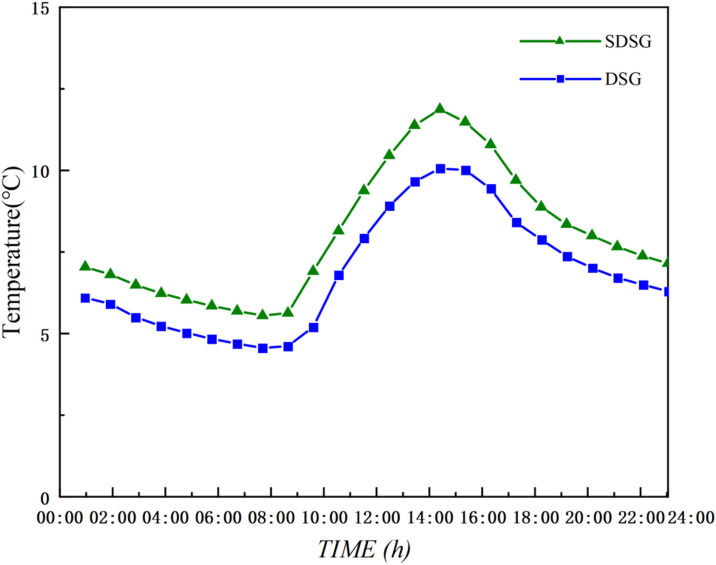
Comparison of simulated shaded section temperatures throughout the day.

A comparative analysis was conducted on the temperature fields of the shaded sections in the DSG and SDSG, with the temperature variation curves shown in Fig 9 (S3 File). The results indicate that the temperature curves of the shaded sections in both the DSG and SDSG follow a consistent trend and maintain a stable difference.

As shown in Fig 9, between 10:00 and 17:00, the average indoor air temperature of the SDSG shaded room is 1.19–1.81°C higher than that of the DSG shaded room; between 18:00 and 9:00 the next day, the average temperature of the SDSG shaded room is 0.88–1.71°C higher than that of the DSG. This is because, compared with the DSG, the sunken shaded room of the SDSG has a larger soil contact area, thereby increasing soil heat storage. At the same time, the sunken shaded room forms a larger space, which increases its overall heat capacity and reduces the ratio of exposed surface area to cold air. Consequently, its insulation performance is further enhanced, allowing the shaded room of the SDSG to maintain a temperature of 6.91–11.87°C throughout the day, meeting the overwintering growth requirements of low-temperature edible fungi (5–13°C) [39].

### Simulation analysis of wall temperatures

The temperature distribution of the wall directly reflects the direction of heat flow [15]. Therefore, in studying the temperature distribution of the walls in the three types of solar greenhouses, cross-sections of the rear wall of the SSG and the central walls of the DSG and SDSG, located at distances of 45, 30, and 15 meters from the east gable wall, were selected as reference points. The simulation analysis was conducted at 24:00, when nighttime temperatures are lower, to reveal the variations and differences in wall temperature distribution along both the north-south and east-west directions.

**Fig 10:**
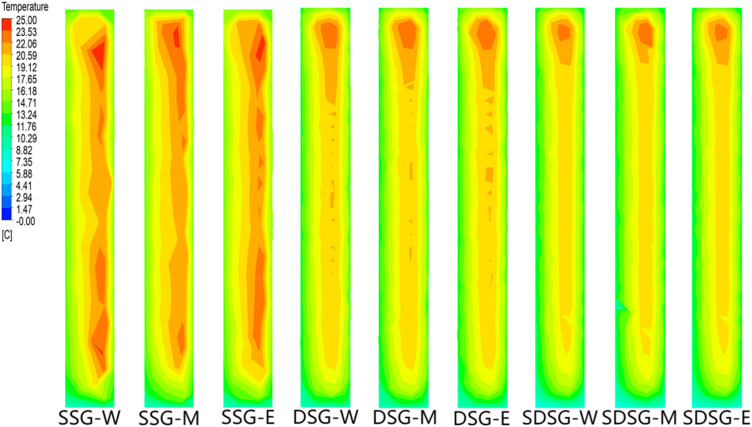
Simulation of wall temperature fields at 24:00 in the three types of solar greenhouses. Note: The right side of the figure represents the south side of the rear wall. W, M, and E denote the cross-sections of the rear wall located 45, 30, and 15 meters from the east gable wall, respectively.

As shown in Fig 10, at 24:00, the wall temperature differences among the three types of solar greenhouses are not very pronounced in the east-west direction. In the north-south direction, however, all exhibit a trend of higher temperatures in the middle section and lower temperatures at both ends. This pattern indicates that, at night, the heat stored in the rear wall of the SSG is released both inside and outside the greenhouse. The central walls of the DSG and SDSG simultaneously release heat to both the shaded and sunlit sections. Additionally, since the shaded section of the SDSG is larger than that of the DSG, more heat must be transferred from the sunlit section to maintain the indoor environment. As a result, there are differences in the heat storage status of the greenhouse walls, with the SSG rear wall storing more heat overall.

**Fig 11:**
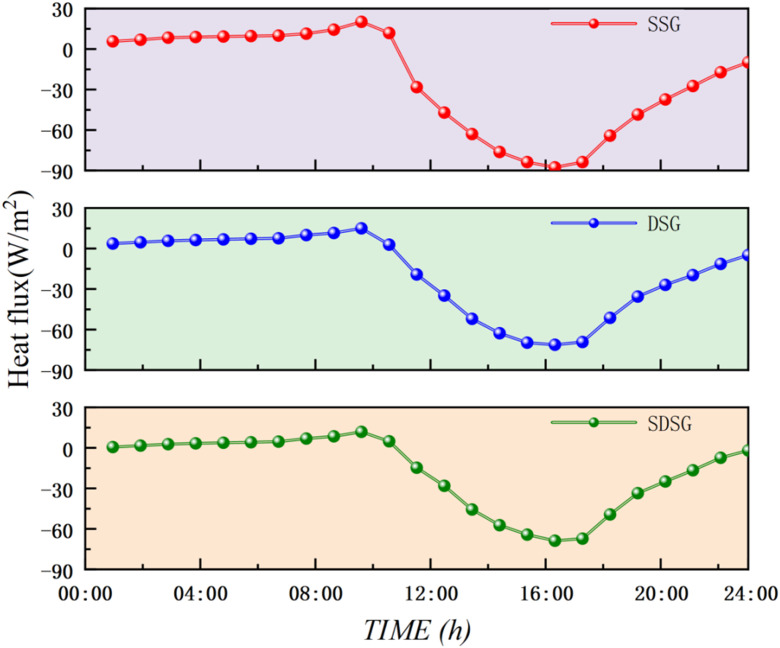
Comparison of simulated wall heat flux densities for the three types of solar greenhouses.

To further understand and clarify the heat storage and insulation capacities of the rear walls in the three types of solar greenhouses, a comparative analysis was conducted on the instantaneous heat flux variations for the rear wall of the SSG and the central walls of the DSG and SDSG. As shown in Fig 11 (S4 File), between 00:00 and 10:00, the heat flux density of the walls is positive, indicating that the rear wall of the SSG and the central walls of the DSG and SDSG are releasing heat into the greenhouse, with heat also flowing indoors. At 9:00, when the insulation quilt is rolled up, the heat flux density of the walls reaches its peak, with maximum values of 20.14, 14.98, and 11.88 W/m², respectively. Afterward, the heat flux density gradually decreases, and the direction of heat flow gradually changes. From 11:00–24:00, the heat flux density of the walls becomes negative, indicating a complete reversal in the direction of heat flow—the walls of all three types of solar greenhouses begin to absorb solar radiation and store heat, while heat also flows from outdoors to indoors.

The heat flux density of the SSG rear wall exhibits the largest variation range, reflecting its role as the primary heat storage body in a single-space structure, bearing the greatest diurnal temperature load. The large temperature difference with the external environment drives intense heat flow. In comparison, the heat flow trends of the central walls in the DSG and SDSG are nearly identical, with their minimum heat flux densities being 16.35 W/m² and 18.81 W/m² higher, respectively, than that of the SSG rear wall. This occurs because the temperature difference between the sunlit and shaded rooms in the DSG and SDSG is smaller than the temperature difference between the SSG and the external environment, indicating that their wall heat storage and release capacities are stronger than those of the SSG. Moreover, the variation in heat flux density of the SDSG central wall is more stable, and the duration of heat release is longer than that of the DSG. This is mainly because the sunken shaded room of the SDSG reduces the exposed surface area of the surrounding soil, allowing the soil and walls to form a synergistic heat storage system that decreases the nighttime heat loss rate, slows down heat release, and prolongs the duration of heat emission.

From the comparison of the daily wall heat flux density curves, it can be concluded that the variation in heat flux density of the SDSG is more stable, and the duration of heat release is longer than that of the DSG. This indicates that the SDSG achieves a smoother and more sustained heat supply, reflecting its superior nighttime insulation performance and higher thermal stability.

### Annual temperature simulation analysis

To comprehensively investigate the adaptability and practical application potential of the SDSG under year-round climatic conditions, three typical solar terms in China were selected—the Spring Equinox (March 21), Summer Solstice (June 22), and Autumn Equinox (September 23)—for simulation analysis of the thermal environments of the three types of solar greenhouses.

**Fig 12:**
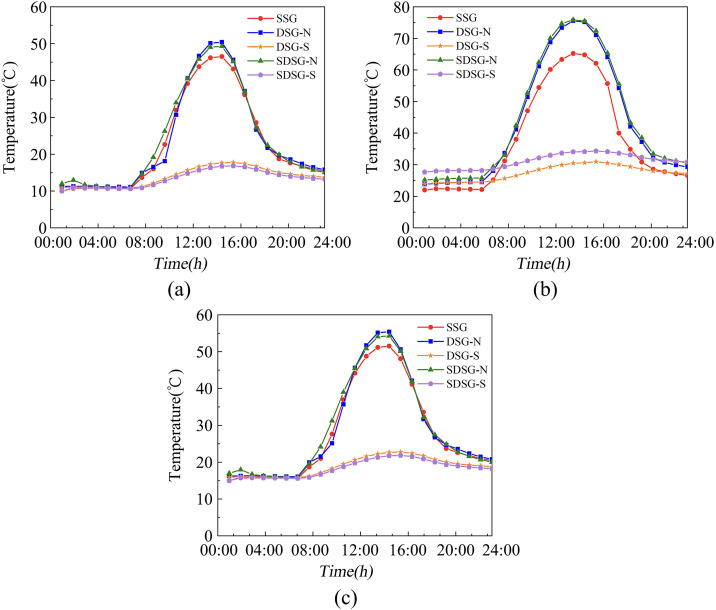
Comparison of simulated daily temperatures for the three types of solar greenhouses across different seasons. (a) Comparison of simulated daily temperatures for the three types of solar greenhouses on March 21 (b) Comparison of simulated daily temperatures for the three types of solar greenhouses on June 22 (c) Comparison of simulated daily temperatures for the three types of solar greenhouses on September 23. Note: N and S denote the sunlit room and the shaded room, respectively.

As shown in Fig 12 (S5 File), the simulation results for spring and autumn (March 21 and September 23) indicate that the diurnal temperature difference in the SDSG is 1.3–1.75°C lower than that in the DSG, and the temperature in the shaded section of the SDSG is 3.38–3.7°C higher than that in the DSG. Since the diurnal temperature difference in spring and autumn is relatively large, the sunken structure effectively buffers external temperature fluctuations through soil heat storage, further improving the temperature environment in the shaded section. The simulation results for summer (June 22) show that the temperature in the shaded sections of both the DSG and SDSG remains between 25 and 30°C, which is conducive to the growth and development of high-temperature edible fungi. In autumn, the shaded section temperatures of the DSG and SDSG range from 15 to 20°C, which is optimal for the growth and development of edible fungi. The seasonal variations in temperature curves demonstrate that the shaded section temperatures of both the DSG and SDSG can extend the suitable production period of edible fungi and at the same time reduce the idle rate of land in solar greenhouses.

### Thermal stability index (TSI)

The Thermal Stability Index (TSI) is commonly used to evaluate the heat resistance of materials [40]. In this study, the concept is extended and applied to solar greenhouses, serving as a core parameter for quantifying the heat storage and buffering performance of solar greenhouses. It reflects the greenhouse’s ability to maintain internal temperature stability under long-term external temperature fluctuations. The thermal stability index is defined as the fluctuation in temperature values inside and outside the greenhouse per unit time. The higher the value, the smaller the temperature fluctuation. The formula is as follows:


$$TSI=\frac{\Delta T_{out}}{\Delta T_{in}}$$ (7)

**Fig 13:**
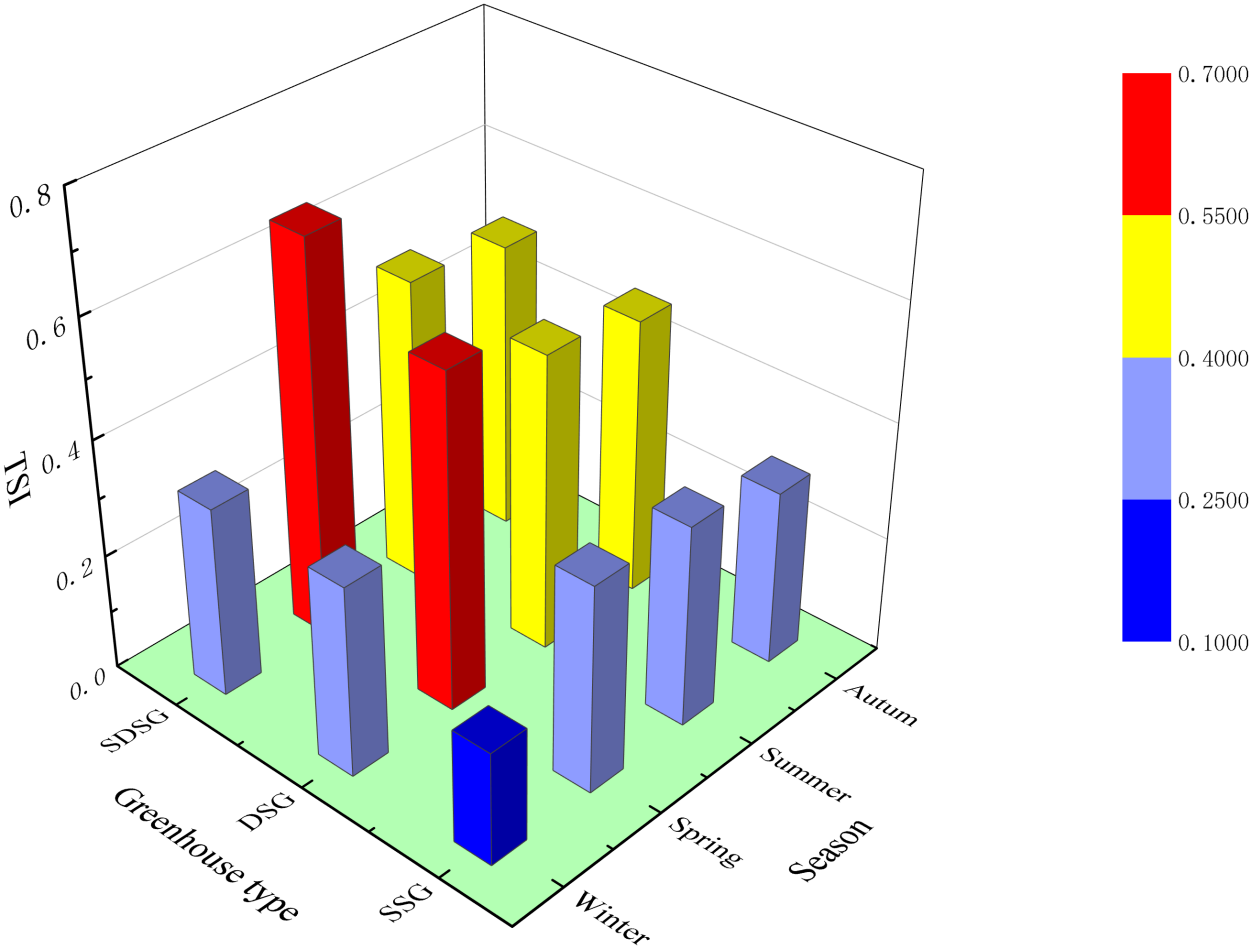
Thermal stability index of solar greenhouses across different seasons.

As shown in Fig 13 (S6 File), the thermal stability index of the SDSG is consistently higher than those of the SSG and DSG across different seasons. The annual average TSI for the SDSG is 0.51, which is 70% and 8.5% higher than those of the SSG (0.30) and DSG (0.47), respectively. This indicates that the SDSG has smaller temperature fluctuations and stronger heat storage and buffering capacity, better meeting the requirements for stable temperature differentials needed for indoor crops [41].

## Conclusions

In this study, CFD software was used to simulate the indoor and wall temperature variations of single-slope, double-slope, and sunken double-slope solar greenhouses. The following conclusions were drawn based on comparative analysis:

1) Using Fluent to establish the physical models and inputting the selected initial external conditions, simulations of the thermal environments for the three different types of solar greenhouses were conducted. Model validation demonstrated a high degree of agreement between simulated and experimental results.
2) In winter, the maximum temperature of the sunlit section in the SDSG is 1.9°C higher than that in the SSG, and the minimum temperature is 2.08°C higher than that in the SSG. The temperature in the shaded section of the SDSG is 0.88–1.81°C higher than that in the DSG, significantly improving the winter thermal environment of the solar greenhouse.
3) In winter, compared to the rear wall of the SSG, the heat flux density of the central wall in the SDSG is 10.19 W/m² lower; it is similar to that of the DSG’s central wall, but the heat release duration in the SDSG is longer than that in the DSG.
4) Throughout the year, the annual average thermal stability index of the SDSG is 0.51, which is 70% and 8.5% higher than those of the SSG (0.3) and DSG (0.47), respectively. This indicates that the SDSG exhibits smaller temperature fluctuations and a stronger capacity for heat storage and buffering.

The findings of this study provide both a theoretical foundation and a practical pathway for improving land utilization efficiency and optimizing the thermal environment for crop growth in solar greenhouses. It should be noted that, in this simulation, the CFD model did not include the effects of indoor crops. However, in real-world production settings, the thermal physiological effects of crops can significantly influence the heat storage and release processes of components such as soil and walls, as well as the convective heat transfer of indoor air. This may potentially affect the model’s accuracy. Moreover, although the consistency between field test results and simulation outcomes validates the model, this study primarily used CFD simulation results as the main basis for comparison. The thermal environment characteristics and practical adaptability of renovated greenhouses still require further research and quantitative assessment.

## Supporting information

S1 File Measured data. (XLS)

S2 File Temperature of the solar room. (XLS)

S3 File Temperature of the shade room. (XLS)

S4 File Heat flux density of the wall. (XLS)

S5 File Greenhouse temperature in March. (XLS)

S6 File Thermal stability index. (XLS)
